# The Stathmin-2 membrane-targeting domain is required for axon protection and regulated degradation by DLK signaling

**DOI:** 10.1016/j.jbc.2023.104861

**Published:** 2023-05-24

**Authors:** Emma J.C. Thornburg-Suresh, Jerianne E. Richardson, Daniel W. Summers

**Affiliations:** 1 Interdisciplinary Graduate Program in Neuroscience, University of Iowa, Iowa City, Iowa, USA; 2 Department of Biology, University of Iowa, Iowa City, Iowa, USA; 3 Iowa Neuroscience Institute, University of Iowa, Iowa City, Iowa, USA

**Keywords:** Stmn2, Stmn3, axon, palmitoylation, DLK, JNK, microtubule, neurodegeneration, membrane trafficking, protein degradation

## Abstract

Axon integrity is essential for functional connectivity in the nervous system. The degeneration of stressed or damaged axons is a common and sometimes initiating event in neurodegenerative disorders. Stathmin-2 (Stmn2) is an axon maintenance factor that is depleted in amyotrophic lateral sclerosis, and replenishment of Stmn2 can restore neurite outgrowth in diseased neurons. However, mechanisms responsible for Stmn2-mediated axon maintenance in injured neurons are not known. We used primary sensory neurons to interrogate the role of Stmn2 in the degeneration of severed axons. We discover that membrane association of Stmn2 is critical for its axon-protective activity. Structure–function studies revealed that axonal enrichment of Stmn2 is driven by palmitoylation as well as tubulin interaction. Using live imaging, we discover that another Stmn, Stmn3, comigrates with Stmn2-containing vesicles. We also demonstrate that Stmn3 undergoes regulated degradation through dual leucine zipper kinase (DLK)–c-Jun N-terminal kinase signaling. The Stmn2 membrane-targeting domain is both necessary and sufficient for localization to a specific vesicle population and confers sensitivity to DLK-dependent degradation. Our findings reveal a broader role for DLK in tuning the local abundance of palmitoylated Stmns in axon segments. Moreover, palmitoylation is a critical component of Stmn-mediated axon protection, and defining the Stmn2-containing vesicle population will provide important clues toward mechanisms of axon maintenance.

Neurons extend long structures called axons that establish connections throughout an organism. In humans, some axons project over a meter in length to reach their targets. This incredible distance poses unique biological challenges for the axon compartment. One such challenge is the transport of cargo through the entire length of the axon to supply nerve terminals with proteins necessary for function. Axonal damage or stress can impair transport and threaten axon integrity. For example, axon transection deprives distal axon segments of the survival factor Nmnat2 and stimulates Sarm1-mediated axon dismantling (1, 2, 3). Loss of axon health is an early sometimes initiating event in a variety of neurodegenerative disorders in the peripheral and central nervous systems (4). Therefore, defining cellular pathways that control axon susceptibility to pathological degeneration is an important need.

The microtubule-binding phosphoprotein Stathmin-2 (Stmn2) is an axon maintenance factor that is depleted in amyotrophic lateral sclerosis (ALS). ALS-linked mutations in TDP-43 cause aberrant splicing of Stmn2 mRNA and decreased Stmn2 protein expression in humans with ALS and frontotemporal lobar degeneration (5, 6, 7, 8, 9). In mouse models, loss of Stmn2 provokes motor and sensory neuropathies (10, 11, 12), and selective knockdown of Stmn2 in dopaminergic neurons leads to neuron loss and locomotor deficits (13). Moreover, replenishing Stmn2 restores neurite outgrowth in induced pluripotent stem cell–derived motor neurons from patients with ALS (5, 6). These discoveries collectively reinforce the important contribution of Stmn2 to axon maintenance.

Stmn2 is a member of the Stmn family of phosphoproteins that include Stmn1, Stmn3, and Stmn4 (14, 15). Proteins in the Stmn family directly bind tubulin heterodimers to regulate microtubule stability. Stmn interaction with tubulin heterodimers is regulated by phosphorylation events in an unstructured proline-rich domain (PrD) present in all Stmn proteins (16). For example, the mitogen-activated protein kinase (MAPK) c-Jun N-terminal kinase (JNK) phosphorylates Stmn2 on serines in the PrD and thereby regulates microtubule dynamics in neurons (17, 18). However, phosphorylation can also impact Stmn2 function beyond tubulin interaction. JNK-mediated phosphorylation of Stmn2 promotes regulated degradation of this protein (19). Alanine substitutions in two serines phosphorylated by JNK boosts local abundance of Stmn2 in axons, and overexpression of this phospho-dead Stmn2 variant delays axon degeneration in transected axons (19). JNK is phosphorylated downstream of the MAP3K dual leucine zipper kinase (DLK), and stimulating DLK accelerates loss of Stmn2 protein from axons (20). Whether the DLK–JNK signaling pathway similarly regulates degradation of other Stmn family members is unknown.

In addition to phosphorylation, Stmn2 is palmitoylated on two cysteine residues in an N-terminal membrane-targeting domain (MTD) (21). These cysteines are labeled with ^3^H-palmitate and required for enrichment on membrane-bound subcellular compartments isolated by biochemical fractionation or visualized under fluorescence microscopy (21, 22, 23). The Stmn2 MTD is necessary and sufficient for localization to the Golgi as well as vesicles in the growth cone (22, 23). Recombinant fragments of Stmn2 lacking the MTD can still bind tubulin heterodimers and regulate microtubule stability *in vitro* (24, 25). Therefore, membrane association is not necessary for tubulin interaction. Palmitoylation is required for Stmn2-dependent regulation of amyloid precursor protein processing and trafficking to the cell surface (26), indicating an important contribution to Stmn2 function in neurons. However, a role for Stmn2 palmitoylation in axon degeneration has not been examined.

We discovered that palmitoylation is required for Stmn2-mediated axon protection. Blocking Stmn2 palmitoylation did not prevent Stmn2 from accumulating in axons as the tubulin-binding region (TBR) also contributes to axonal localization. A highly related MTD is present in Stmn3 and functionally compensates for the corresponding MTD in Stmn2. Moreover, Stmn2 and Stmn3 comigrate on the same vesicle population. Consistent with behavior observed for Stmn2, Stmn3 is short lived, rapidly lost from transected axons, and undergoes regulated degradation in response to DLK–JNK signaling. Notably, Stmn1 lacks an MTD and does not display these features. The Stmn2 MTD is sufficient to localize a reporter protein to Stmn2-containing vesicles and confer DLK-dependent instability in axon segments. Collectively, these observations identify new functional roles for membrane association in Stmn2-mediated axon integrity.

## Results

### Stmn2-mediated axon protection requires palmitoylation

Stmn2 is regulated by two different post-translational modifications, phosphorylation, and palmitoylation. Serine-to-alanine replacement of JNK phosphorylation sites (Stmn2AA) enhances Stmn2 axon-protective activity (19). Protein levels of Stmn2AA were also elevated because of extended half-life in axon segments suggesting that prolonged stability is responsible for axon protection. Stmn2 protein turnover is also regulated by palmitoylation (27), yet a functional role for this post-translational modification in axon degeneration has not been examined. To address this question, we converted two cysteine residues modified by palmitoylation to serines (Stmn2CS) and evaluated how the loss of palmitoylation impacts Stmn2 activity in Wallerian degeneration, a model of pathological axon loss.

**Figure 1:**
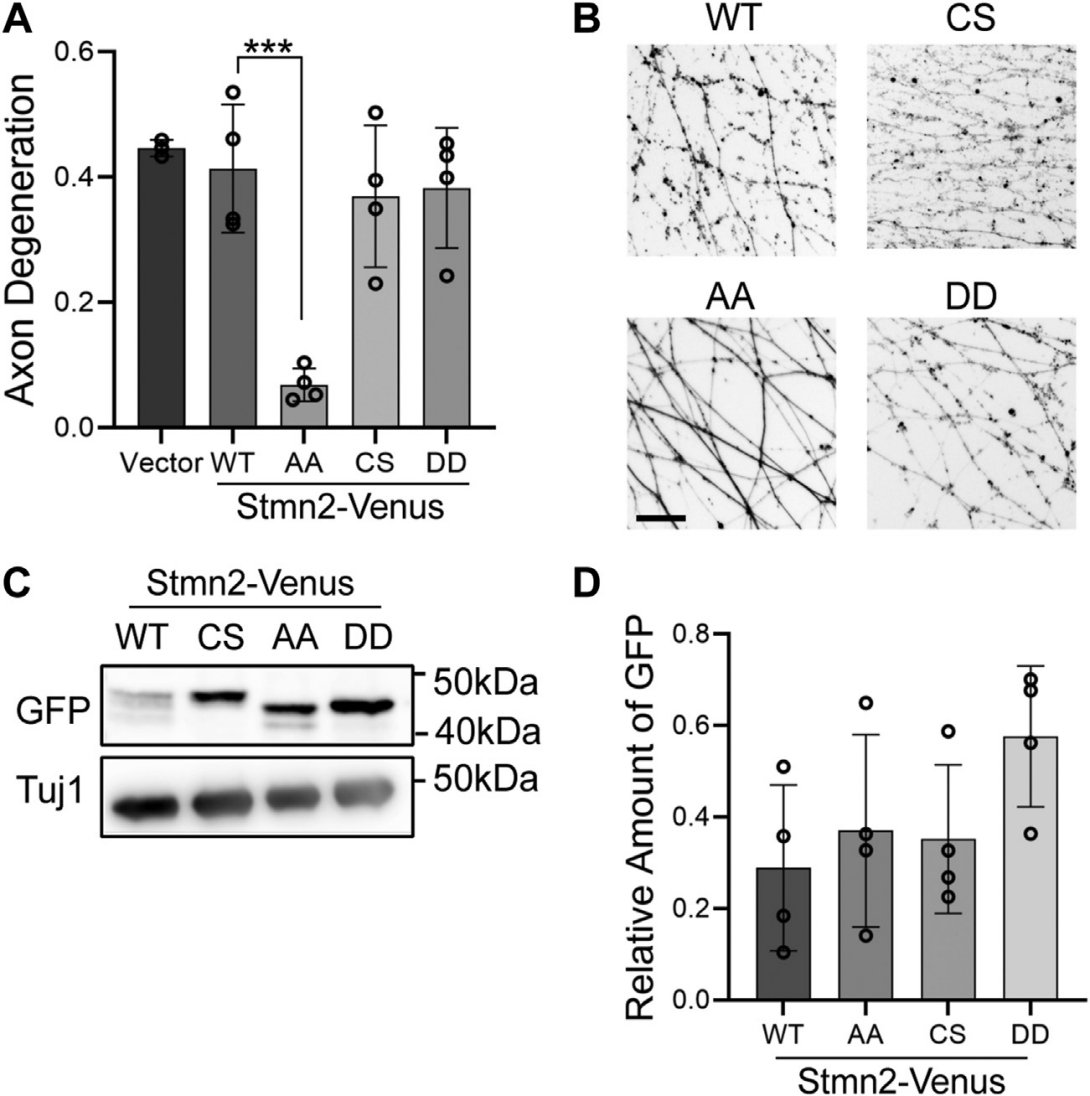
**Palmitoylation is required for Stmn2-mediated axon protection.***A*, DRG sensory neurons were transduced with lentiviruses expressing the indicated human, Venus-tagged Stmn2 constructs, and axons were severed with a razor blade. The degeneration of distal axons was measured 10 h post axotomy (WT; CS C22,24S; AA S62,73A; DD C22,24S, and S62,73A). Axon degeneration values were subjected to a one-way ANOVA with Bonferroni post hoc *t* test (*F*(4, 14) = 12.63, *p* = 0.0001, WT *versus* AA ∗∗∗*p* = 0.005, n = 4). *B*, representative images of distal axons 10 h post axotomy labeled with myristoylated mRuby3. *C*, Western blot of Venus-tagged Stmn2 variants from axon-only extracts with quantification shown in (*D*) (n = 4). Differences in Stmn2 variant protein levels were not statistically significant (one-way ANOVA, *F*(3, 12) = 1.947, *p* = 0.1758). Scale bar represents 5 μm. Error bars represent ±1 STD. DRG, dorsal root ganglia; Stmn, stathmin.

We overexpressed wildtype or mutant forms of Stmn2 tagged with Venus *via* lentiviral transduction in embryonic-derived mouse sensory neurons from dorsal root ganglia (DRG). For all experiments, we used a Stmn2 human coding sequence that shares 100% amino acid homology with mouse Stmn2. Three days following lentiviral transduction with Stmn2 expression constructs, axons were cut with a razor blade to induce degeneration of severed axon segments. We replicated past findings that overexpression of Stmn2AA delayed axon degeneration 10 h post axotomy (Fig. 1, *A* and *B*). Overexpression of Stmn2CS behaved similar to wildtype Stmn2 and did not affect axon degeneration (Fig. 1, *A* and *B*). To assess whether palmitoylation is necessary for axon protection evoked by Stmn2AA, we generated a version of Stmn2 lacking both JNK phosphorylation and palmitoylation (Stmn2; double dead; DD). In contrast to Stmn2AA, Stmn2DD did not suppress axon degeneration 10 h post axotomy (Fig. 1, *A* and *B*), indicating that palmitoylation is required for Stmn2AA-mediated axon protection.

We suspected that loss of palmitoylation would interfere with axonal transport of Stmn2, which may account for the lack of axon-protective activity observed from the Stmn2DD construct. We examined protein levels of each Stmn2 construct in axons by Western immunoblotting. As previously observed (19, 27), Stmn2AA and Stmn2CS protein levels were increased in axon-only extracts as compared with wildtype Stmn2. Notably, Stmn2DD protein levels were further increased compared with wildtype, Stmn2AA, and Stmn2CS (Fig. 1 *C* and *D*). Despite abundant levels of Stmn2DD protein in the axon segment, this Stmn2 variant did not delay axon degeneration suggesting that palmitoylation is required for Stmn2-mediated protection of severed axons.

### The TBR contributes to enrichment of Stmn2 in axons

**Figure 2:**
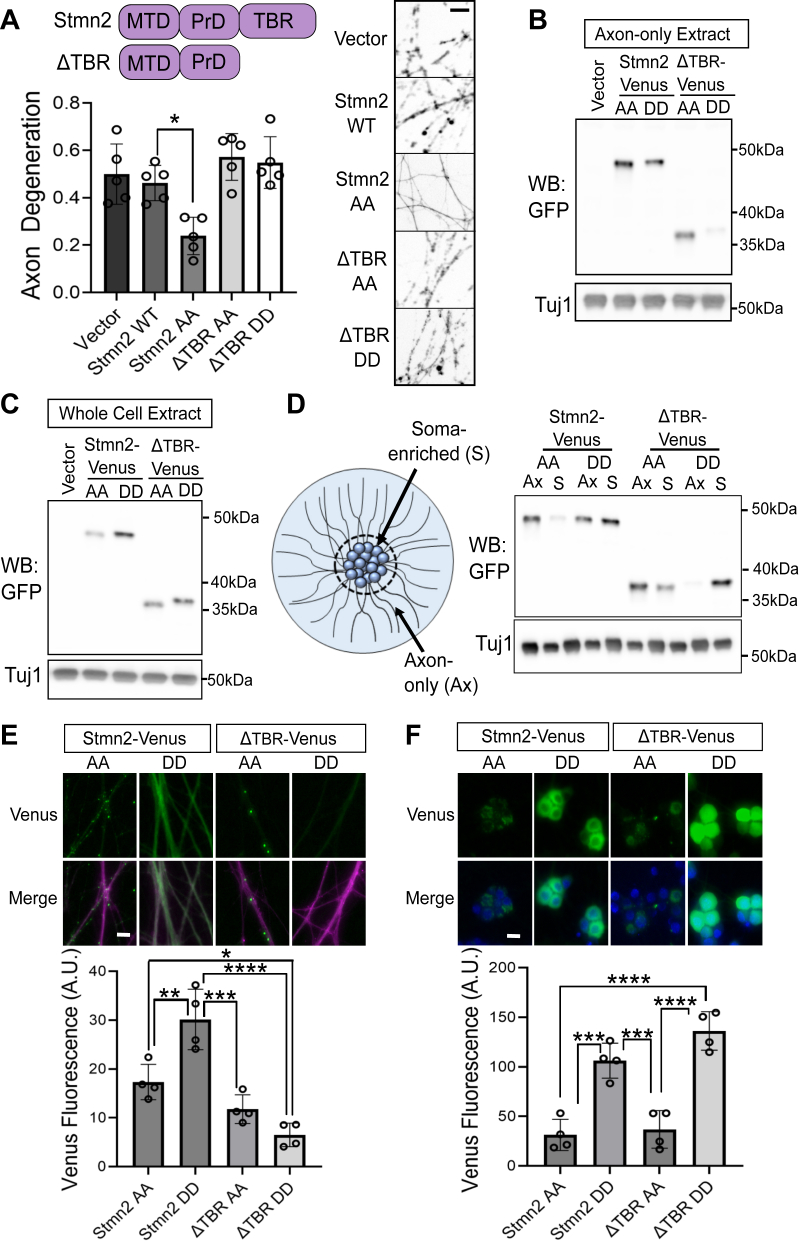
**The tubulin-binding region (TBR) participates in Stmn2 localization and axon protection.***A*, domain structure of Stmn2 identifying the membrane-targeting domain (MTD), Stmn-like/proline-rich domain (PrD), and TBR. We measured axon degeneration 10 h after axotomy in the presence of the indicated Stmn2 constructs (full length or ΔTBR; AA S62,73A; DD C22,24S and S62,73A) (one-way ANOVA with Bonferroni post hoc *t* tests; *F*(4, 20) = 8.92, *p* = 0.0003, n = 5). Representative images of distal axons labeled with myristoylated mRuby3 at 10 h post axotomy shown to the right. Scale bar represents 5 μm. Western immunoblot detection of Venus-tagged Stmn2 constructs from (*B*) axon-only extracts or (*C*) whole-cell extracts. *D*, axon-only and soma-enriched fractions were collected from the same culture expressing the indicated Stmn2-Venus construct and detected together by Western immunoblot. *E*, fluorescence microscopy of distal axons from DRG sensory neurons expressing the indicated Stmn2-Venus construct and myristoylated mRuby3 to label axons (*magenta*). Quantification of Venus fluorescence shown below (n = 4). *F*, fluorescence microscopy of Venus expression in the soma of DRG sensory neurons and merged image with nuclei labeled with Hoechst. Quantification of fluorescence shown below (n = 4). For (*E*) and (*F*), means were compared with one-way ANOVA, and statistical results from Bonferroni post hoc *t* tests are shown in the graphs (panel *E*: *F*(3, 12) = 33.12, *p* < 0.0001 and panel *F*: *F*(3, 12) = 25.16, *p* < 0.0001). Error bars represent ±1 STD. Differences that reach statistical significance are identified (∗*p* < 0.05, ∗∗*p* < 0.01, ∗∗∗*p* < 0.001, ∗∗∗∗*p* < 0.0001). Scale bar represents 20 μm. DRG, dorsal root ganglia; Stmn, stathmin.

Stmn2 possesses an MTD, an Stmn-like and PrD (designated together here as PrD), and TBR (Fig. 2*A*) (14). We demonstrated a functional role for Stmn2 palmitoylation in the MTD for suppressing axon degeneration (Fig. 1); however, a role for the Stmn2 TBR has not been evaluated. To address this question, we expressed a version of Stmn2AA lacking the TBR (ΔTBR-AA) as well as a version that also lacked palmitoylation (ΔTBR-DD) and measured axon degeneration 10 h after axotomy. Expressing ΔTBR-AA did not suppress axon degeneration indicating the TBR is required for Stmn2-mediated axon protection (Fig. 2*A*).

Since expressing ΔTBR-AA did not suppress axon degeneration, we wanted to confirm that Stmn2 fragments used in Figure 2*A* were localizing to axon segments. We observed equivalent levels of ΔTBR-AA compared with full-length forms of Stmn2 in axon-only extracts. However, axonal levels of ΔTBR-DD were reduced compared with full-length Stmn2AA, Stmn2DD, and ΔTBR-AA (Fig. 2*B*). When we examined whole-cell extracts, ΔTBR-DD protein levels were comparable to other Stmn2 constructs (Fig. 2*C*). Based on these findings, we speculated that ΔTBR-DD was retained in the soma. Axon-only and soma-enriched protein fractions were isolated from the same culture and directly compared by Western immunoblotting (Fig. 2*D*). Stmn2-AA and ΔTBR-AA seemed to be predominantly detected in the axon-only fraction, whereas Stmn2-DD was detected in both axon-only and soma-enriched fractions, consistent with a predicted role for palmitoylation in vesicle association and anterograde transport. However, ΔTBR-DD was largely restricted to the soma-enriched fraction (Fig. 2*D*). We also quantified levels of Venus-tagged Stmn2 fragments in cell bodies and axons under fluorescence microscopy (Fig. 2, *E* and *F*). Nonpalmitoylated full-length Stmn2-Venus (FL-DD) was increased in axon segments; however, removing the TBR (ΔTBR-DD) reversed this effect, and the protein was localized almost exclusively to the soma. In the presence of palmitoylation, removing the TBR did not cause a significant change in axonal enrichment. These data indicate that tubulin interaction contributes to axonal localization of nonpalmitoylated forms of Stmn2. Moreover, the TBR is required for Stmn2-mediated axon protection after axotomy.

### The MTD and PrD of Stmn2 are required for axon protection

**Figure 3:**
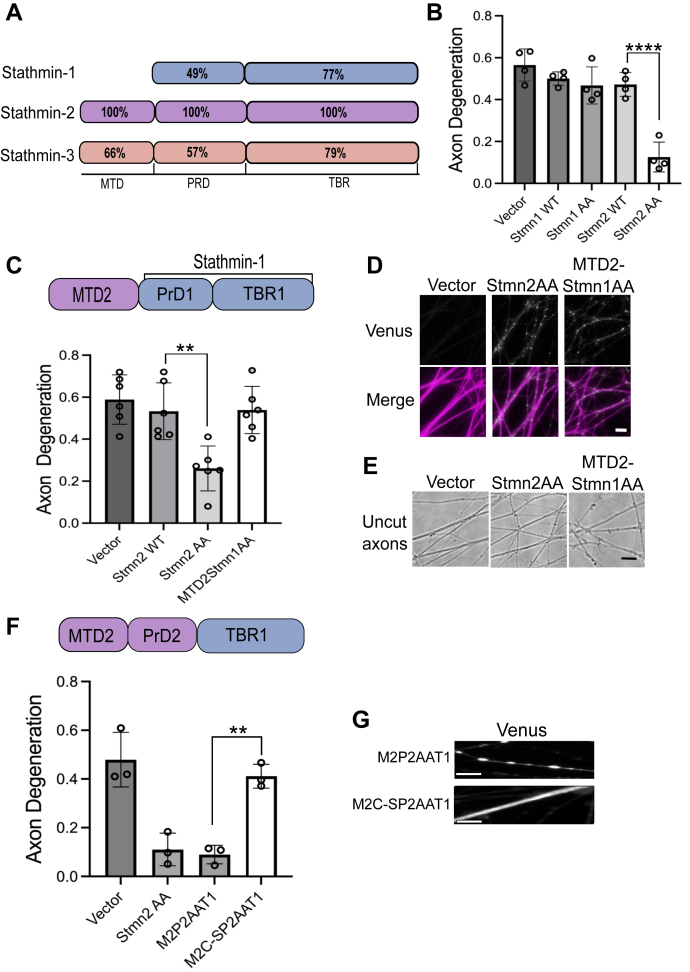
**The Stmn1 tubulin-binding region (TBR) functionally compensates for the TBR of Stmn2 in axon protection.***A*, domain structures of Stmn1, Stmn2, and Stmn3 with percent homology displayed relative to Stmn2. *B*, degeneration of transected axons from DRG sensory neurons expressing the indicated Stmn construct (n = 4, one-way ANOVA with Bonferroni post hoc *t* tests, *F*(4, 15) = 25.8, *p* < 0.0001). *C*, axon degeneration 10 h after axotomy from DRG sensory neurons expressing Stmn2AA or MTD2-Stmn1AA compared with an empty vector (n = 6, one-way ANOVA with Bonferroni post hoc *t* test, *F*(4, 25) = 9.383, *p* < 0.0001). *D*, MTD2-Stmn1AA-Venus is localized to puncta and observed in axons labeled with mRuby3 (*magenta*). *E*, phase contrast images of uncut distal axons. Axonal blebbing is apparent in DRGs expressing MTD2-Stmn1AA-Venus. Scale bar in (*D*) and (*E*) equals 15 μm. *F*, axon degeneration in DRG sensory neurons 10 h post axotomy expressing Venus-tagged Stmn2AA or a Stmn2 AA chimera with the Stmn1 TBR (M2P2AAT1). We also evaluated axon degeneration in the presence of a M2P2AAT1 construct with mutations that impair palmitoylation (M2C-P2AAT1). Each construct was compared with the empty vector control (n = 4, one-way ANOVA with Bonferroni post hoc *t* test, F3, 8 = 23.24, *p* = 0.0003). *G*, Venus-tagged Stmn2 chimeras were localized to distal axons. Differences that reach statistical significance are identified (∗∗*p* < 0.01, ∗∗∗∗*p* < 0.0001). Error bars represent ±1 STD. Scale bar represents 5 μm. DRG, dorsal root ganglia; MTD, membrane-targeting domain; Stmn, stathmin.

In contrast to the other Stmns, Stmn1 does not possess an MTD and is not palmitoylated (28) (Fig. 3*A*). Overexpression of wildtype, human, Stmn1 did not suppress axon degeneration 10 h post axotomy (Fig. 3*B*). We generated alanine substitutions in Stmn1 at serine 25 and serine 38 (Stmn1AA), which are phosphorylated by JNK (29) and correspond to the alanine substitutions in Stmn2AA (30). In contrast to Stmn2AA, overexpression of Stmn1AA did not suppress axotomy-induced axon degeneration (Fig. 3*B*).

Since palmitoylation is required for Stmn2-mediated axon protection, we predicted that adding the Stmn2 MTD to Stmn1AA would endow this Stmn1 construct with axon-protective activity. We generated a construct wherein the MTD of Stmn2 (amino acids 1–40) was placed on the N terminus of full-length Stmn1AA (MTD2Stmn1AA). However, in contrast to Stmn2AA, overexpression of MTD2Stmn1AA did not suppress axon degeneration (Fig. 3*C*). MTD2Stmn1AA was localized to puncta in axon segments (Fig. 3*D*); however, overexpression of MTD2Stmn1AA resulted in baseline toxicity prior to axotomy, as indicated by axonal blebbing (Fig. 3*E*), and confounded our ability to evaluate this question.

We next generated a construct that incorporates both the MTD and PrD of Stmn2 (containing alanine substitutions at Ser62 and Ser73) with the tubulin-binding domain of Stmn1 (M2P2AAT1). Overexpression of M2P2AAT1 suppressed axon degeneration to a similar extent as Stmn2AA (Fig. 3*F*). Based on our findings in Figure 1, we predicted that activity of this chimera would be dependent on palmitoylation. Indeed, cysteine to serine substitutions that block palmitoylation (M2C-SP2AAT1) abrogated axon-protective activity (Fig. 3*F*). Fluorescence microscopy confirmed that both constructs are present in distal axon segments, and preventing palmitoylation results in diffuse localization (Fig. 3*G*). Consequently, the Stmn2 MTD and PrD can function with the Stmn1 TBR to suppress axon degeneration.

### Stmn3 possesses axon-protective activity that is palmitoylation dependent

**Figure 4:**
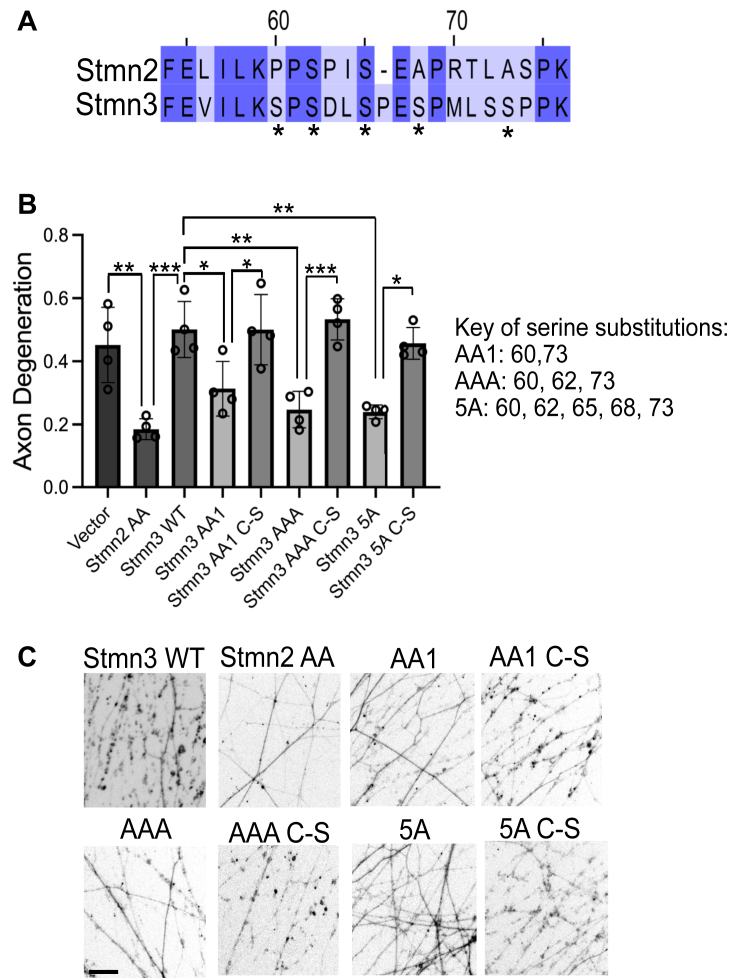
**Stmn3 displays axon-protective effects that are palmitoylation dependent.***A*, sequence alignment of the proline-rich domain (PrD) from Stmn2 and Stmn3. *Asterisks* identify serine residues in Stmn3 that are modified in this study. *B*, degeneration of transected axons from DRG sensory neurons expressing the indicated Stmn constructs. A key describing the Stmn3 alanine-replacement constructs is shown on the *right*. Each Stmn3 construct was also generated with amino acid substitutions that impair palmitoylation indicated by a C-S. Phosphorylation-dead constructs are compared with its matched palmitoylation-dead construct (n = 4, one-way ANOVA with Bonferroni post hoc *t* tests, *F*(8, 27) = 12.01, *p* < 0.0001). *C*, representative images of distal axons 10 h post axotomy. Error bars represent ±1 STD (∗*p* < 0.05, ∗∗*p* < 0.01, ∗∗∗*p* < 0.001, ∗∗∗∗*p* < 0.0001). Scale bar represents 5 μm. DRG, dorsal root ganglia; Stmn, stathmin.

Within the Stmn family, Stmn2 shares the most homology with Stmn3. We predicted that interfering with JNK-phosphorylated serines in the Stmn3 PrD would generate an axon-protective protein. The PrD of Stmn2 and Stmn3 share 57% homology (Fig. 4*A*); however, the Stmn3 PrD is phosphorylated at additional serines in this region by different kinases (31). We generated serine to alanine replacements in numerous residues previously identified as substrates for phosphorylation. These Stmn3 variants were expressed in DRGs, and the degeneration of severed axons was measured 10 h post axotomy. *In vitro* studies with Stmn3 identify Ser60 as the predominant JNK target, and Ser73 corresponds to the serine in Stmn2 predominantly phosphorylated by JNK (30). An alanine replacement in Ser60,73 (Stmn3 AA1) conferred modest axon protection when overexpressed (Fig. 4, *B* and *C*).

We generated an additional alanine replacement at Ser62, which corresponds to a modified serine in Stmn2AA based on sequence alignment to generate a triple alanine mutant (Stmn3 AAA). We also generated an Stmn3 variant with five alanine replacements (Stmn3 5A) at putative JNK phosphorylation sites in the PrD. All three Stmn3 variants displayed enhanced axon protection (Fig. 4, *B* and *C*). Stmn3 AAA and Stmn3 5A displayed axon protection comparable to Stmn2 AA in severed axons at 10 h post axotomy (Fig. 4, *B* and *C*). Stmn3 is palmitoylated (21, 32), and we predicted that interfering with this post-translational modification would reverse the axon-protective activity of these Stmn3 proteins. We generated cysteine to serine substitutions in the Stmn3 MTD of all three Stmn3 variants. Cysteine to serine substitutions in the Stmn3 MTD that prevent palmitoylation also suppressed the axon-protective activity of these Stmn3 alanine variants (Fig. 4, *B* and *C*). Therefore, similar to Stmn2, palmitoylation is required for Stmn3-mediated axon protection.

### Stmn2 and Stmn3 comigrate in sensory neuron axons

**Figure 5:**
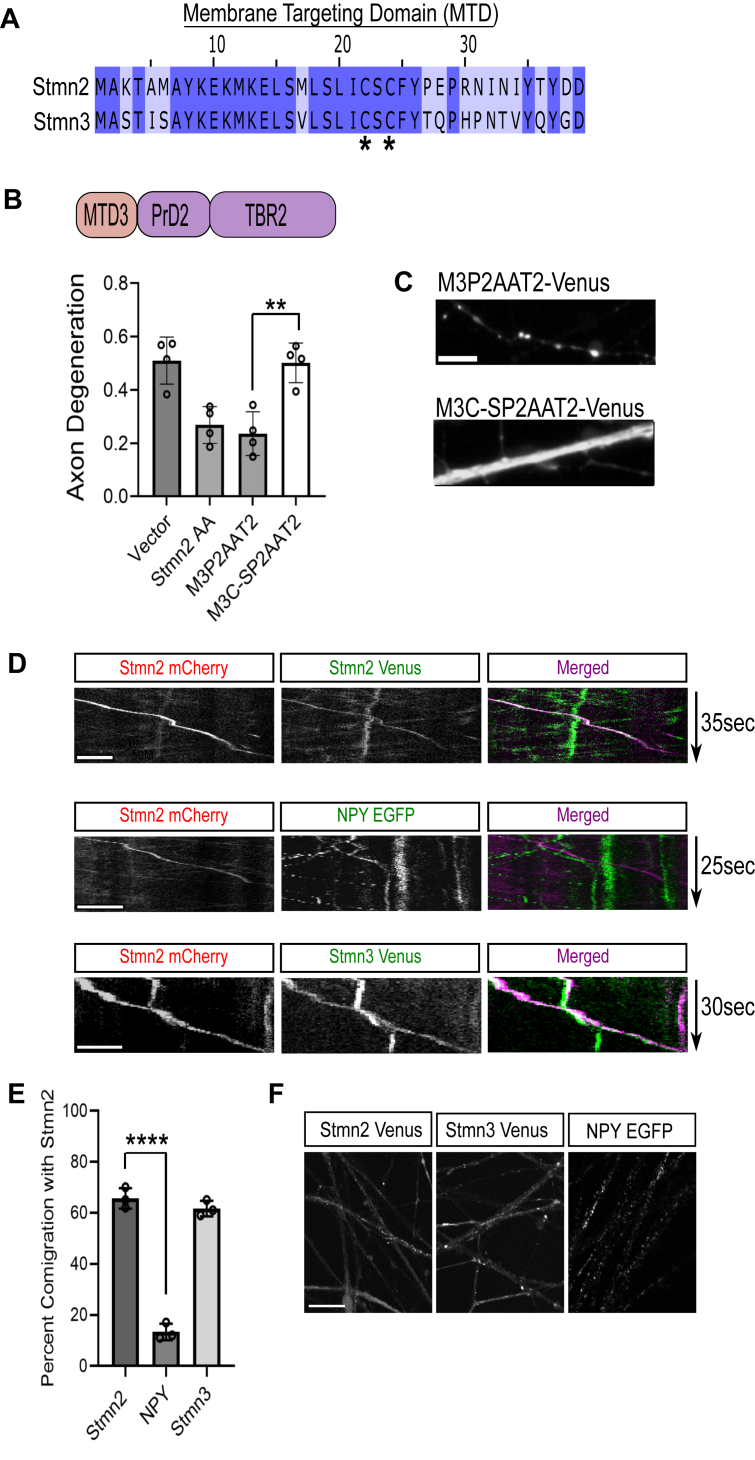
**Stmn2 and Stmn3 comigrate in sensory neuron axons.***A*, sequence alignment of the membrane-targeting domain (MTD) of Stmn2 and Stmn3. *Asterisks* indicate palmitoylation sites. *B*, degeneration of transected axons from DRG sensory neurons 10 h postaxotomy expressing Venus-tagged Stmn2 AA or indicated Stmn2AA chimera with the MTD of Stmn3 (M3P2AAT2). We also evaluated axon degeneration in the presence of chimeras with mutations that impair palmitoylation (M3C-SP2AAT2) (n = 4, one-way ANOVA with Bonferroni post hoc *t* tests, *F*(3, 12) = 13.93, *p* = 0.0003). *C*, localization of Venus-tagged M3P2AAT2 and M3C-SP2AAT2 in axon segments. Scale bar represents 5 μm. *D*, representative kymographs from live-imaging studies following comigration between Stmn2-mCherry puncta with either Stmn2-Venus (*top panels*), NPY-EGFP (*middle panels*), or Stmn3-Venus (*bottom panels*) in axon segments. Grayscale kymographs are included for individual channels with merged kymograph to show puncta comigration. Scale bars in (*C*) and (*D*) represent 5 μm. *E*, quantification of comigration with Stmn2 (n = 3, unpaired *t* test). Error bars represent ±1 STD. ∗∗*p* < 0.01, ∗∗∗∗*p* < 0.0001. *F*, representative images of axons from DRG sensory neurons expressing Stmn2-Venus, Stmn3-Venus, or NPY-EGFP. There are abundant NPY-EGFP vesicles present in axon segments despite low comigration with Stmn2. Scale bar represents 10 μm. DRG, dorsal root ganglia; EGFP, enhanced GFP; Stmn, stathmin.

The MTDs from Stmn2 and Stmn3 share considerable homology (66% identity) (Fig. 5*A*) and undergo palmitoylation at two cysteine residues within this domain. To evaluate whether these domains can functionally compensate for one another, we swapped the MTD of Stmn2 with the MTD of Stmn3 in the presence of the axon-protective Stmn2 AA substitution (M3P2AAT2). Expression of this chimeric protein suppressed axon degeneration to a similar extent as Stmn2-AA at 10 h post axotomy (Fig. 5*B*). Blocking palmitoylation with cysteine to serine substitutions in the Stmn3 MTD (M3C-P2AAT2) reversed this axon-protective activity. Wildtype and palmitoylation-dead versions of this chimera were both localized in axons; however, the palmitoylation-dead version was diffuse (Fig. 5*C*).

Since the MTD of Stmn3 functionally compensates for the MTD of Stmn2, we hypothesized that Stmn2 and Stmn3 are targeted to the same vesicle population. We transduced differentially tagged versions of Stmn2 and Stmn3 constructs into DRG sensory neurons and used live imaging to track comigration. To assess maximal comigration we expect from our assay, we first cotransduced mCherry and Venus-tagged versions of Stmn2 and measured the rate of comigration in axon segments. From this analysis, we observed 65% comigration between Stmn2-containing particles (Fig. 5, *D* and *E*). Next, we evaluated comigration between Stmn2-mCherry and Stmn3-Venus particles in axon segments. We observed 62% comigration between Stmn2-mCherry and Stmn3-Venus labeled particles, which is comparable to our comigration measurements with differentially tagged Stmn2 proteins. To assess the specificity of this assay, we measured comigration between Stmn2-mCherry with neuropeptide Y (NPY). NPY is trafficked through secretory vesicles that are predicted to have lower rates of comigration with Stmn2-mCherry compared with Stmn3. We observed 13% comigration between Stmn2-mCherry and NPY-enhanced GFP (EGFP), suggesting minimal comigration between these two proteins (Fig. 5, *D* and *E*). Despite low comigration between NPY-EGFP and Stmn2-mCherry puncta, we observe abundant NPY-EGFP puncta in distal axons (Fig. 5*F*). Moreover, NPY-EGFP largely resides in discrete puncta within axons, whereas Stmn2-Venus and Stmn3-Venus display a mixed pattern that includes both discrete puncta as well as diffuse localization. Therefore, Stmn2 and Stmn3 comigrate on the same vesicle population, and the MTDs are functionally equivalent in axon maintenance.

### The MTD of Stmn2 promotes DLK-dependent degradation

**Figure 6:**
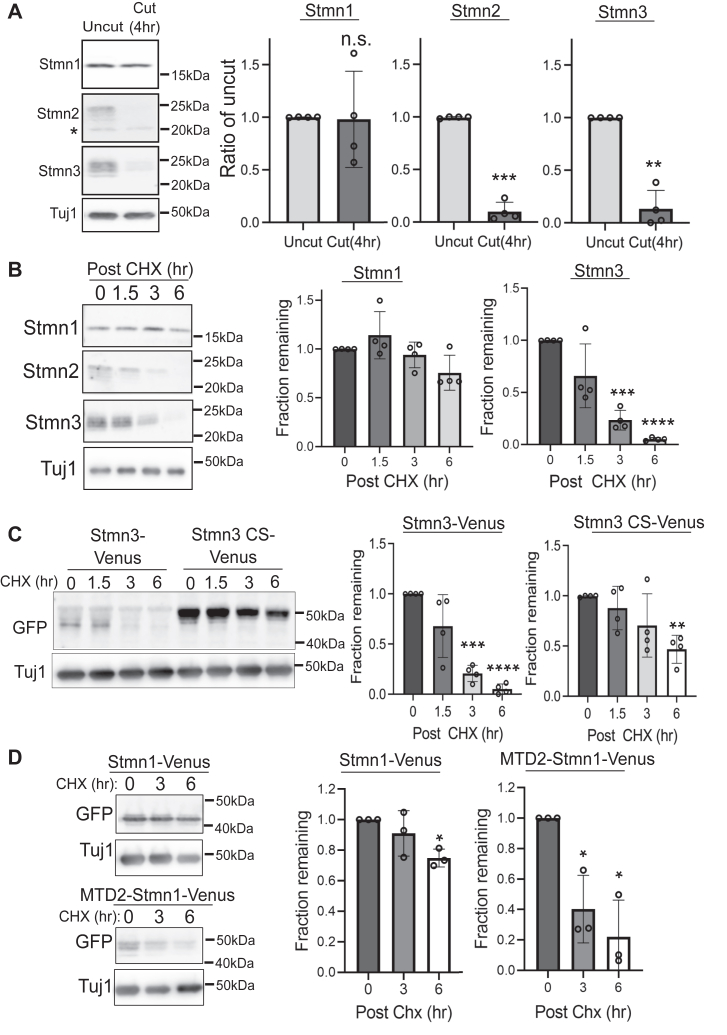
**Membrane-associated Stathmins (Stmn) are targeted for degradation.***A*, Western immunoblot analysis of endogenous Stmn1, Stmn2, and Stmn3 from intact or severed axons 4 h post cut (n = 4, unpaired Welch’s *t* test; ns = not significant). ∗indicates nonspecific band detected by Stmn2 antibody. *B*, protein turnover of endogenous Stmn1, Stmn2, and Stmn3 following addition of cycloheximide (CHX, 25 μg/mL) to inhibit protein synthesis for the indicated times. Each time point was compared with the 0 h control (n = 4, unpaired Welch’s *t* test). *C*, protein turnover of Venus-tagged Stmn3 constructs (CS; C22,24S) detected *via* Western immunoblotting following CHX addition (25 μg/mL). Each time point is compared with the 0 h control (n = 4, unpaired Welch’s *t* test). *D*, protein turnover of Venus-tagged Stmn1 (Stmn1-Venus) and membrane-targeted Stmn1 (MTD2-Stmn1-Venus) detected *via* Western immunoblotting following addition of CHX (25 μg/mL). Each time point is compared with the 0 h control (n = 3, unpaired Welch’s *t* test). Representative Western blots are shown to the *left*. Error bars represent ±1 STD (∗<0.05, ∗∗<0.01, ∗∗∗<0.001, ∗∗∗∗<0.0001). MTD, membrane-targeting domain.

Our experiments identify a critical role for membrane localization and palmitoylation in Stmn2 axon-protective function in severed axons. Palmitoylation also contributes to regulated turnover of Stmn2 protein (27). Stmn2 is a short-lived protein, and axon transection leads to depletion of Stmn2 protein from severed axons in DRG sensory neurons (19). To determine if this property is unique to Stmn2, we evaluated whether Stmn1 and Stmn3 are depleted in axons after axotomy. At 4 h post axotomy, protein levels of Stmn2 and Stmn3 were reduced below 10% of uncut control (Fig. 6*A*). Stmn1 protein levels were unchanged compared with uncut control.

We next evaluated the half-life of endogenous Stmn1 and Stmn3 in severed axon segments. We inhibited protein synthesis with cycloheximide (CHX) and monitored loss of endogenous Stmn1, Stmn2, and Stmn3 in axon-only extracts by Western immunoblotting. The half-life of Stmn3 in axons was approximately 2 h (Fig. 6*B*), similar to the half-life of Stmn2 (19). In contrast, Stmn1 protein levels were unchanged at 3 h post CHX treatment and reduced by only 20% after 6 h of CHX treatment. We examined whether palmitoylation contributes to Stmn3 turnover as observed in Stmn2 (27). We compared protein turnover rates between wildtype Stmn3-Venus with a version that is not palmitoylated (Stmn3CS-Venus). The half-life of Stmn3CS-Venus was extended to 6 h (Fig. 6*C*) supporting a role for palmitoylation in protein turnover of both Stmn2 and Stmn3 in axons.

Stmn1 is not associated with membranes and displays greater stability in axon segments compared with Stmn2 and Stmn3. We attached the Stmn2 MTD to the N terminus of Stmn1 to ascertain whether the Stmn2 MTD could confer instability on another Stmn. Stmn1-Venus or MTD2-Stmn1-Venus were expressed in DRGs, and protein turnover was evaluated in axon-only extracts after CHX treatment. Consistent with our measurements of endogenous Stmn1, Venus-tagged Stmn1 decreased approximately 20% after 6 h of CHX treatment (Fig. 6*D*). On the other hand, MTD2-Stmn1-Venus protein levels decreased 80% by 6 h post-CHX addition indicating the Stmn2 MTD can confer instability on another Stmn.

**Figure 7:**
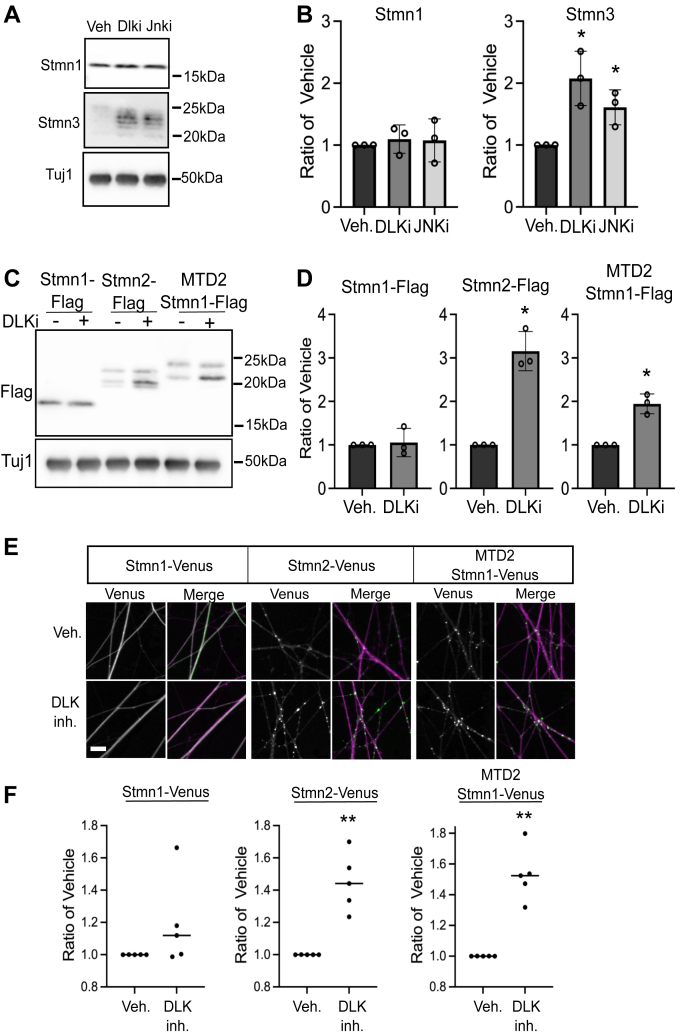
**Membrane association targets Stathmins (Stmns) for DLK-dependent degradation.***A*, representative Western blots of endogenous Stmn1 and Stmn3 following acute treatment with DLK and JNK inhibitors (1 μM GNE-3511 and 5 μM JNK inhibitor VIII) for 4 h with quantification shown in (*B*), drug treatment was compared with vehicle control (n = 3, unpaired Welch’s *t* test). *C*, representative Western blot of FLAG-tagged Stmn1, Stmn2, and membrane-targeted Strmn1 (MTD2 Stmn1) following treatment with vehicle or DLK inhibitor (DLKi, 1 μM GNE-3511) (4 h) with quantification of FLAG-positive bands in (*D*) (n = 3, unpaired Welch’s *t* test). *E*, representative images of Venus-tagged Stmn1, Stmn2, and MTD2 Stmn1 treated with vehicle or DLKi (4 h, 1 μM GNE-3511). Merge images show overlay between Venus and myristoylated mRuby3 (*magenta*) used to label axons. Quantification is shown in (*F*) (n = 5, unpaired Welch’s *t* test). Error bars represent ±1 STD (∗*p* < 0.05, ∗∗*p* < 0.01). Scale bar represents 20 μm. DLK, dual leucine zipper kinase; JNK, c-Jun N-terminal kinase.

MAPK signaling *via* the DLK–JNK pathway promotes regulated turnover of palmitoylated Stmn2 (27). Whether DLK signaling regulates axonal levels of other Stmn proteins is not known. Acutely blocking DLK or JNK activity with small-molecule inhibitors (1 μM GNE-3511 and 5 μM JNK inhibitor VIII) for 4 h increased steady state, axonal levels of endogenous Stmn3 yet did not affect endogenous Stmn1 (Fig. 7, *A* and *B*). Since adding the Stmn2 MTD to Stmn1 accelerated its turnover, we evaluated whether the Stmn2 MTD confers sensitivity to DLK signaling. For these experiments, expression constructs were generated with a FLAG epitope. Using this smaller epitope allowed us to resolve differentially modified species of Stmn2 protein that are also detected in endogenous Stmn2 (17, 18, 19). We detected multiple FLAG-positive Stmn2 bands, whereas Stmn1-FLAG resolved as a single band. GNE-3511 treatment did not affect Stmn1-FLAG levels in axon-only extracts. However, GNE-3511 treatment did induce a significant increase in Stmn2-FLAG, and MTD2-Stmn1-FLAG protein levels, particularly a lower molecular weight protein species (Fig. 7, *C* and *D*). As a complementary approach, we used Venus-tagged versions of these constructs to quantify changes in Venus fluorescence intensity in distal axon segments (Fig. 7*E*). Stmn1-Venus fluorescence intensity levels were not affected by GNE-3511. However, GNE-3511 treatment induced a significant increase in Stmn2-Venus and MTD2-Stmn1-Venus fluorescence intensity (Fig. 7*F*).

### The Stmn2 MTD is sufficient to promote vesicle comigration and DLK-dependent degradation

**Figure 8:**
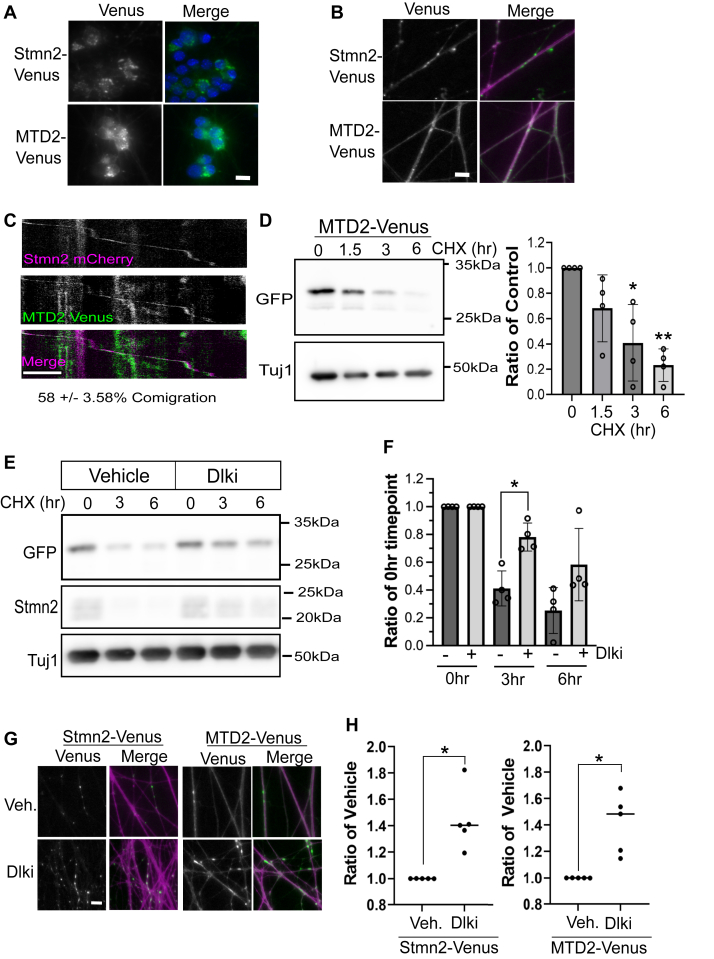
**The membrane-targeting domain (MTD) is sufficient for vesicular targeting and regulation by DLK.** Representative images of Venus-tagged full-length Stmn2 (Stmn2-Venus) and just the MTD (MTD2-Venus). Images are merged with either Hoechst to stain for the cell body (*A*) or myristoylated mRuby3 to label axons (*B*) (scale bar represents 10 μm). *C*, representative kymographs of Stmn2 mCherry and MTD2 Venus showing comigration (scale bar represents 5 μm). Comigration of MTD2 with Stmn2 determined to be 58 ± 3.58% (n = 3, unpaired *t* test). *D*, levels of the Venus-tagged MTD of Stmn2 (MTD2) were detected *via* Western immunoblotting following treatment with cycloheximide (CHX, 25 μg/mL) for the indicated period. Each time point was compared with the 0 h control (n = 4, unpaired Welch’s *t* test). Representative Western blot shown to the *left*. *E*, representative Western blot of Venus-tagged MTD2 following acute treatment with vehicle or DLK inhibitor (DLKi, 1 μM GNE-3511), and treatment with cycloheximide (CHX, 25 μg/mL) at indicated periods. Endogenous Stmn2 shown as a control for DLK treatment. Quantification of MTD2-Ven turnover treated with vehicle or DLKi is shown in (*F*). Vehicle and DLKi are compared for each time point post CHX treatment (two-way ANOVA with Bonferroni post hoc *t* tests, n = 4). *G*, representative images of Venus-tagged Stmn2 and MTD2 following treatment with vehicle or DLKi (1 μM GNE-3511). With quantification shown in (*H*), DLKi compared with the vehicle (n = 5, unpaired Welch’s *t* test). Scale bar represents 20 μm. Error bars represent ±1 STD. Differences that reach statistical significance are identified (∗*p* < 0.05, ∗∗*p* < 0.01). DLK, dual leucine zipper kinase; Stmn, stathmin.

Attaching the MTD from Stmn2 to GFP relocalizes this fluorescent protein to Golgi as well as vesicles in the growth cones of hippocampal neurons (33). The authors of this study also observed colocalization between this GFP fusion and endogenous Stmn2. We attached the Stmn2 MTD to Venus and evaluated localization, comigration with Stmn2, and protein turnover in DRG sensory neurons. MTD2-Venus localized to puncta in the soma and axon, similar to full-length Stmn2 (Fig. 8, *A* and *B*). We also observed a stronger diffuse MTD2-Venus population in both axons and soma compared with Stmn2-Venus. Focusing on the population of axonal vesicles, we measured comigration between MTD2-Venus with full-length Stmn2-mCherry. We observed 58% comigration between MTD2-Venus with Stmn2-mCherry (Fig. 8*C*), which is comparable to the level of comigration between differentially tagged forms of Stmn2 (Fig. 5).

Considering differential stability between Stmn2 and Stmn1 and the impact of adding the Stmn2 MTD to Stmn1, we next determined whether the Stmn2 MTD is sufficient to promote regulated degradation of a protein other than an Stmn. We measured turnover of MTD2-Venus in axon-only extracts after CHX treatment. MTD2-Venus levels decreased by 30% after 1.5 h CHX treatment and 80% by 6 h (Fig. 8*D*). Since Stmn2 degradation is regulated by DLK signaling, we next examined whether MTD2-Venus degradation was dependent on this pathway. In the presence of the DLK inhibitor GNE-3511, the turnover kinetics of the MTD2 were slowed, with only a 20% decrease after 3 h CHX treatment and 40% drop by 6 h (Fig. 8, *E* and *F*). We also examined steady-state levels in axons with fluorescence microscopy in response to DLK inhibition. Axonal MTD2-Venus levels increased to a comparable level as full-length Stmn2-Venus after 4 h GNE-3511 treatment (Fig. 8, *G* and *H*). We conclude that the Stmn2 MTD promotes localization to a specific Stmn2-containing vesicle population and confers regulated degradation *via* the DLK signaling.

## Discussion

Stmns are a family of phosphoproteins with a long-appreciated function in microtubule dynamics. While heavily studied in the context of neurite outgrowth, Stmns also contribute to axon maintenance. Stmn1^−/−^ mice develop late-onset axonopathy (34), and loss of Stmn2 is linked to motor neuropathy in mice and humans (5, 6, 7, 8, 10, 11). In particular, re-expressing Stmn2 in ALS-derived human motor neurons rescues axon outgrowth defects *in vitro* (5, 6). Therefore, restoring Stmn2 protein in axon segments is a potential therapeutic opportunity; however, mechanisms required for Stmn2 axon-protective activity are not clear. We demonstrate that palmitoylation is required for Stmn2-mediated axon protection. Notably, blocking Stmn2 palmitoylation did not prevent Stmn2 enrichment within the axon segment. Instead, tubulin interaction through the TBR also promotes Stmn2 localization to axons suggesting the relationship between Stmns and the microtubule cytoskeleton is bidirectional. Stmns regulate microtubule dynamics by sequestering tubulin heterodimers while also using the microtubule network for transport into distal axon segments.

In contrast to other members of the Stmn family, Stmn1 does not possess an MTD and did not display inherent axon-protective activity in severed axons. Attaching the Stmn2 MTD to Stmn1 triggered spontaneous axonal blebbing and did not confer axon-protective activity. In contrast, introducing the Stmn2 MTD and PrD on the Stmn1 TBR was well tolerated and generated an axon-protective Stmn protein. There might be intramolecular interactions between the MTD and PrD that influence Stmn function in a cell. The PrD is an unstructured region present in all Stmns, yet also displays the highest divergence in amino acid sequence (14). Moreover, relocalizing Stmn1 to a vesicle might interfere with normal protein–protein interactions on the Stmn2 vesicle that led to toxicity.

The identity of the Stmn2 vesicle population is not known. Stmn3 comigrates with Stmn2 in axon segments suggesting there are vesicles enriched with Stmn proteins. Stmn3 is also phosphorylated by JNK (30), and alanine substitutions in the Stmn3 PrD conferred axon-protective activity similar to Stmn2AA. The Stmn3 MTD can functionally compensate for the Stmn2 MTD, and palmitoylation is required for Stmn3 axon-protective activity. Whether palmitoylation affects interaction between Stmn2 and tubulin heterodimers is not known. Palmitoylation is dispensable for tubulin binding *in vitro*; however, other residents on the Stmn2 vesicle could influence this interaction inside the cell. Stmn2 palmitoylation also contributes to its function in protein trafficking of amyloid precursor protein and chromaffin (26, 35). Moreover, Stmn2 regulates mitochondrial transport in axons (19) indicating a broader, still unclear function in organelle transport that could have significant relevance to axon maintenance and dysfunction in disease.

**Figure 9:**
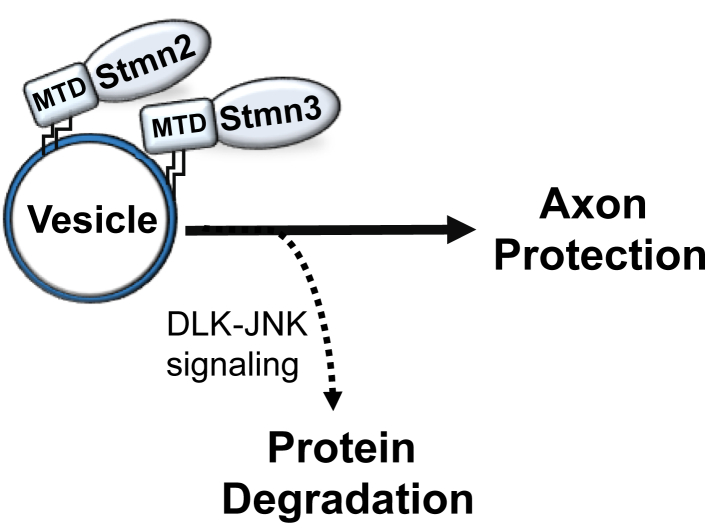
**Working model for regulated degradation of membrane-associated Stathmins (Stmn) by DLK–JNK signaling.** The membrane-targeting domains (MTDs) on Stmn2 and Stmn3 are palmitoylated and promote localization to a specific vesicle population. Membrane association is required for axon protection afforded by these Stmns. DLK–JNK signaling promotes regulated degradation of these membrane-associated Stmns and rapidly tunes local Stmn abundance in response to cellular conditions. The mechanism in which DLK signaling drives degradation of palmitoylated proteins on this vesicle is unknown. DLK, dual leucine zipper kinase; JNK, c-Jun N-terminal kinase.

The regulated degradation of palmitoylated Stmns by an MAPK pathway enables both rapid and local depletion of Stmn protein within an axon segment. Conversely, anterograde transport will replenish the local Stmn population when this MAPK pathway is inactive. Elevation and depletion of Stmn abundance will have opposing consequences on microtubule dynamics and axon integrity within the axon segment (Fig. 9). Alanine replacements in JNK-phosphorylated serines extend Stmn2 stability suggesting that direct phosphorylation in the PrD promotes degradation (19). However, attaching the Stmn2 MTD alone to a fluorescent reporter shortens half-life and confers sensitivity to DLK–JNK signaling even in the absence of the Stmn2 PrD. JNK-mediated phosphorylation of the Stmn2 MTD is not reported, yet cannot be ruled out. This MTD promotes comigration with Stmn2 on the same vesicle population and might be targeted for degradation by association. These comigration studies required attaching a genetically encoded fluorescent protein to Stmn2 and Stmn3. The presence of a fluorescent tag partially extends Stmn2 half-life; however, these fusion proteins are still regulated by DLK–JNK signaling (19). Excess Stmn2 protein might saturate endogenous trafficking pathways and lead to partial mislocalization to new membrane compartments or accumulate as nonpalmitoylated species. Interestingly, many proteins in the DLK–JNK signaling complex are palmitoylated, including DLK itself (36, 37). DLK signaling is activated in response to microtubule dysfunction (38, 39, 40) raising the possibility that DLK is a conduit between the microtubule cytoskeleton and Stmn2 activity. Understanding how microtubules, DLK, and Stmns communicate in this signaling circuit will generate important insight on mechanisms of axon maintenance.

## Experimental procedures

### Reagents and plasmids

Human Stmn expression clones were from Genscript (STMN1-OHu14092D, STMN2-OHu14465D, and STMN3-OHu10050D), and Gibson cloning was used to generate lentiviral expression plasmids. All lentiviral expression plasmids are under control of the human ubiquitin promoter. Phenol-red free neurobasal, B27 supplement, l-glutamine, penicillin–streptomycin, and Opti-MEM were from Gibco. Nerve growth factor (2.5S) was from Bio-Rad. Laminin was from Corning, and poly-d-lysine (PDL) was from MP Biomedicals. Human embryonic kidney 293T cells were cultured in Dulbecco's modified Eagle's medium (4.5 g/l glucose; Corning) supplemented with heat-inactivated fetal bovine serum (Corning), 2 mM l-glutamine, and penicillin–streptomycin (10 U/ml). Western immunoblotting was performed with the following primary antibodies: anti-GFP (Thermo Fisher; Research Resource Identifier [RRID]: AB_221569; 1:1000 dilution), anti-Stmn1 (Cell Signaling; RRID: AB_2798284; 1:1000 dilution), anti-FLAG (Cell Signaling, RRID: AB_2572291; 1:1000 dilution). anti-Stmn2 (R&D Systems; RRID: AB_10972937; 1:1000 dilution), anti-Stmn3 (Proteintech; RRID: AB_2197399; 1:1000 dilution), anti-Tuj1 (BioLegend; RRID: AB_2562570; 1:10,000 dilution). Detection of primary antibodies was performed with the following secondary antibodies that were visualized with a LI-COR Odyssey imager (Thermo Fisher; RRID: AB_1965956, RRID: AB_2556622 and Cell Signaling Technology; RRID: AB_330924). GNE-3511 and JNK inhibitor VIII were from Cayman Chemicals.

### Isolation and culture of DRG sensory neurons

Embryonic sensory neurons derived from DRG were isolated from embryonic day (E) 13.5 mouse pups (equal number of male and female embryos). For all studies, pregnant CD1 mice were purchased from Charles River Laboratories. DRG sensory neurons were dissociated with trypsin and seeded as droplets in tissue culture–treated plastic dishes precoated with PDL and laminin. DRG neurons were cultured in phenol red–free Neurobasal medium supplemented with 2 mM glutamine, 10 U/ml penicillin–streptomycin, 2% B27 supplement, 50 ng/ml nerve growth factor, and 1 μM 5-fluoro-2′-deoxyuridine/1 μM uridine to inhibit growth of non-neuronal cells. Fresh culture media were added every 2 to 3 days. For axon degeneration experiments, DRG neurons were infected 2 days after plating (day *in vitro* [DIV] 2) with lentiviral particles expressing myristoylated-mRuby3 (myr-mRuby3) to label axon membranes. DRGs were also transduced with lentiviruses that express Bcl-xL to prevent nonspecific toxicity from the overexpression of our lentiviral constructs. Bcl-xL overexpression does not affect the kinetics of axon degeneration in Wallerian degeneration (41). On DIV 2 to 4, lentiviruses containing Stmn expression constructs were applied to neurons. All experimental protocols in this study using mice were reviewed and approved by the University of Iowa Office of the Institutional Animal Care and Use Committee.

### Lentiviral preparation and transduction

To produce lentiviral particles, human embryonic kidney 293T cells were transfected with plasmids expressing vesicular stomatitis viral G protein, the lentiviral packaging plasmid psPAX2, and a lentiviral expression plasmid derived from the FCIV backbone (ubiquitin promoter) (42). Two to 3 days post-transfection, media containing the lentiviral particles were collected, centrifuged for 1 min at 500*g* to remove debris, and the supernatant was used to transduce DRG cultures on DIV 2 to 4.

### Measurement of axon degeneration

Axon degeneration was measured as previously described (43). For axotomy studies, DRGs were seeded in 24- or 96-well plates. On DIV 6 or DIV 7, axons were manually severed from the cell body with a razor blade. Fluorescent images of severed axons were acquired with a Cytation 5 automated microscope (Agilent). Within one experimental replicate, at least three wells were assessed per condition, and within each well, six independent fields were collected. Independent DRG sensory neuron cultures are used for each experimental replicate. For time-course studies, the same axon fields were imaged for the duration of the time series. Axon degeneration was scored from a custom-generated ImageJ macro (National Institutes of Health) (43) that scores fragmented/circular axon segments as a ratio of total axon area for each image. The macro assigns each image a number between 0 and 1 with a higher value representing greater axon degeneration.

### Biochemical analysis of axon-only protein extracts

For analysis of protein levels in axon-only fractions, DRGs were densely seeded in a spot culture in a 12-well plate coated with PDL/laminin with fresh media added every 2 days. On DIV 7 or DIV 8, a razor blade was used to cut around the cell bodies, and the spot of cell bodies removed with a pipette. Axons were washed with PBS and then lysed in radioimmunoprecipitation assay buffer (50 mm Tris–HCl [pH 7.4], 1 mM EDTA, 1% Triton X-100, 0.5% sodium deoxycholate, 0.1% sodium dodecyl sulfate, 150 mM NaCl, 1 mM phenylmethylsulfonyl fluoride, 1× EDTA-free protease inhibitor cocktail [Genesee Scientific], and phosphatase inhibitors 5 mM NaF and 1 mM NaVO_4_). Axon-only extracts were precleared by centrifugation (at 4 °C, 2500*g* for 5 min), and then the supernatant was mixed with sample buffer (65.2 mM Tris–HCl [pH 6.8], 2% SDS, 10% glycerol, 8% beta-mercaptoethanol, and 0.025% bromophenol blue). The samples were boiled before analysis by SDS-PAGE and Western immunoblotting. For analysis of protein turnover, the DRG neurons are treated with 25 μg/ml CHX to inhibit protein synthesis. CHX was added on DIV 6 or DIV 7. The time points for CHX addition are indicated per experiment. Lysates were analyzed by SDS-PAGE and Western immunoblotting for the indicated protein and normalized to levels of tubulin using Tuj1. All quantification of Western immunoblotting is performed with ImageJ.

### Comigration imaging and analysis

For analysis of comigration, neurons are sparsely plated in three to four spots on 35 mm plates (Fluorodish; World Precision Instruments) coated with PDL/laminin. Lentiviruses expressing differentially tagged constructs (Venus or mCherry) were added on DIV 3 or DIV 4. Cultures that displayed axonal blebbing or other signs of degeneration were not used. Images were collected with a LEICA TCS SP8 Confocal Microscope using a 63× oil immersion lens (numerical aperture = 1.20) and resonant scanner. The 35 mm plates are placed into an Okolab incubation chamber, and neurons were maintained at 37 °C, 5% CO_2_, and 20% O_2_ for the duration of the experiment. Images were collected simultaneously in both the red and green channels as well as brightfield to identify axon segments. At least three independent experimental replicates were completed per condition.

Analysis of comigration is conducted in a manner to ensure the experimenter is blinded to the imaging conditions. Prior to analysis of a mobile puncta, the axon is checked to ensure that both viruses are expressed. A mobile puncta is identified in one of the two fluorescent channels, and a kymograph is generated for that particle using the ImageJ Kymograph Builder and merged with a kymograph generated from the corresponding channel. Merged kymographs and individual tracts are scored as comigrating or not. The kymographs are scored for comigration by at least two laboratory members. Between 75 and 100 puncta are analyzed per experimental condition. Percentage of comigration with Stmn2 is calculated per-experimental condition.

### Fluorescence microscopy analysis of Stmn-Venus levels in axons

DRG sensory neurons seeded in 96-well plates were transduced with lentiviruses on DIV 3 to express the indicated Stmn and myr-mRuby3 to label axons. On DIV 6, DRGs were fixed in 4% paraformaldehyde and stored in phosphate-buffered saline. Fixed axons were visualized with a Leica DMi8 inverted fluorescence microscope using a 20× objective (numerical aperture = 0.4) maintaining equivalent settings during image acquisition across experimental replicates. Images were captured with a Leica DFC7000T and processed with ImageJ. Thresholding of myr-mRuby3 axon images was performed to generate axon region of interests (ROIs) for each image. We used rolling ball background subtraction on Venus images and measured fluorescence intensity of the Venus image within the corresponding axon ROI. For each experimental replicate, at least three independent wells were used per condition, and at least two fields were collected per well. To measure fluorescence intensity in the soma, we used rolling ball background subtraction on all Venus images and then measured mean fluorescence intensity within the soma using a standard ROI.

### Statistics

All statistical analyses were performed with GraphPad Prism (GraphPad Software, Inc). Specific statistical tests applied for each experiment are identified in the corresponding figure legend.

## Data availability

All data are contained within the text of this article.

## Conflict of interest

The authors declare they have no conflicts of interest with the contents of this article.
